# Cone beam computed tomography analysis of anterior open bite management using clear aligners: a single-arm retrospective study

**DOI:** 10.1038/s41598-025-09157-x

**Published:** 2025-07-11

**Authors:** Qawas Arwa, Alturki Ghassan, Alsulaimani Fahad, El-Bialy Tarek, Shankargouda Patil, Baeshen Hosam

**Affiliations:** 1 Department of Orthodontics, Faculty of Dentistry, King Abdulaziz University, Jeddah, Saudi Arabia; 2 Division of Orthodontics, Department of Dentistry and Dental Hygiene, School of Dentistry, University of Alberta, Edmonton, Canada; 3 College of Dental Medicine, Roseman University of Health Sciences, South Jordan, UT USA

**Keywords:** Anterior open bite, Vertical control, Clear aligners, CBCT, Malocclusion, Cone-beam computed tomography

## Abstract

Lateral cephalograms have the inherent drawback of superimposition of bilateral structures, while landmarks are more reproducible on CBCT scans. Yet no studies in the literature have utilized 3D imaging to investigate the effects of clear aligners on anterior open bite. Therefore, The aim is to measure the skeletal and dental changes that contribute to anterior open bite closure with clear aligner therapy on CBCT scans. It is a single-arm retrospective study that included 40 cases of anterior open bite who were treated using Invisalign. Pre- and post-treatment CBCT scans were traced to record 13 dental and 3 skeletal measurements. A paired t-test was conducted to compare the mean values of pre- and post-treatment measurements. Combined intrusion of the maxillary right and left molars was statistically significant, meanwhile mandibular molars maintained their vertical position. Maxillary incisors were extruded and retroclined significantly, whereas mandibular incisors were only extruded. While anterior facial height was decreased insignificantly, both lower anterior facial height and mandibular plane angle showed a significant decrease. Clear aligner (Invisalign) therapy is effective in the management of anterior open bite through vertical control, maxillary molars intrusion, maxillary incisors extrusion, maxillary and mandibular incisors retroclination, and mandibular autorotation.

## Introduction

Anterior open bite (AOB) is a malocclusion of the maxillary and mandibular incisors when they do not overlap vertically, with varying degrees of severity^1^. This malocclusion adversely affects an individual’s quality of life by hindering and impairing essential oral functions such as decent mastication and speech, as well as compromising facial aesthetics. Managing AOB poses challenges in diagnosis and treatment planning due to its multifactorial etiology, high propensity to relapse—as vertical growth is the last to cease^2^—and the extrusive nature of conventional fixed appliance. This underscores the importance of treatment planning with the objective of vertical control to prevent undesirable posterior extrusion. Traditionally, AOB has been addressed using a range of orthodontic and orthopedic modalities, including high-pull headgear, posterior bite blocks, and fixed appliance supplemented with auxiliaries such as elastics and temporary anchorage devices (TADs)^3^.

With recent advancements in orthodontics, clear aligner therapy—particularly Invisalign—has emerged as a promising alternative option for managing AOB, as it provides sufficient vertical control owing to the full occlusal coverage^4^. It was theorized that clear aligners could produce a “bite-block” effect to intrude the molars^5–7^. However, studies show mixed findings regarding the precise mechanism underlying bite closure. Some research suggests that Invisalign primarily closes the bite through incisor extrusion^8^, while others report molar intrusion and mandibular autorotation in addition to extrusion of the incisors^4,9^. A retrospective comparison between clear aligners and fixed appliances aided by auxiliaries found no notable differences in molar vertical control^10^.

These studies investigating AOB have predominantly relied on two-dimensional lateral cephalograms, with inherent considerable drawbacks such as superimposition of bilateral structures, and different magnification between the right and left sides. Moreover, patients with facial asymmetry or imperfect head positioning could increase tracing errors^11^. Moreover, patients with facial asymmetry or imperfect head positioning could increase tracing errors. Landmark identification is more reproducible on CBCT scans^12–14^, and the magnification error is eliminated^15^. Yet, to our knowledge, CBCT has not yet been employed as a three-dimensional imaging modality to evaluate the effects of clear aligners.

Therefore, this study aims to evaluate and quantify on CBCT scans the dental and skeletal changes that contribute to anterior bite closure using clear aligner therapy, by testing the following null hypotheses: (1) There is no difference in the pre- and post-treatment molar vertical position. (2) There is no difference in the pre- and post-treatment linear and angular measurements of the incisors. (3) There is no difference in the pre- and post-treatment skeletal measurements.

## Materials and methods

### Sample

This study followed a retrospective cohort design and received ethical approval from the King Abdulaziz University Research Ethical Committee (IRB #075-03-23). The data used were obtained from patients who had signed treatment consent forms that included permission for the use of their de-identified data for research purposes. Therefore, informed consent to participate in this study was waived by the King Abdulaziz University Research Ethical Committee. All methods were performed in accordance with the Declaration of Helsinki and the institutional policies of King Abdulaziz University.

G*Power 3.1 software (Kiel University, Germany) was used to perform sample size calculation^16^. Considering the vertical position of the molar as the primary outcome, the effect size was set at 0.5, according to the results by Khosravi et al.^8^, this was counted as the minimal clinically important difference to detect a shift of 0.5 mm ± 1.0 mm in the molar vertical position. The required sample size was one group of 34 subjects when the power was set at 80% and the alpha error at 0.05.

Routine acquisition of CBCT scans before and after treatment is not standard in clinical orthodontics due to radiation dose considerations, and obtaining multiple groups for comparison without compromising the sample size was not feasible^17^. The subjects were retrospectively retrieved from a single center of an expert orthodontist and Invisalign (Align Technology, Santa Clara, CA, USA) provider. CBCT scans were acquired using i-CAT 17-19DX system (Imaging Sciences International, Hatfield, PA, USA) with an isotropic voxel size of 0.3 mm³. Treatment modality included only the use of attachments for the planned molar intrusion, while the ClinCheck prescription for IPR, expansion, and elastics was individualized for each case.

An initial screening of the complete patient dataset was done to include Invisalign cases with anterior open bite, which was defined by edge-to-edge bite or negative overbite. The selected cases were then reviewed for the following exclusion criteria: missing pre- or post-treatment CBCT scan, patients with syndromes, treatment involving extraction, TADs, surgery, or other appliances in conjunction with Invisalign.

The retrieved records were accessible only to the investigators and included the following: 1—de-identified pre-treatment (T1) and post-treatment (T2) CBCT scans, as Digital Imaging and Communications in Medicine (DICOM) files, 2—patient’s age, 3—patient’s gender, 4—Angle classification, 5—treatment duration. Each set of scans was given a code corresponding to the patient’s data in an Excel sheet dataset.

### Processing and data collection (cephalometric tracing)

DICOM files were reoriented to standardize head position and converted into Nearly Raw Raster Data (NRRD) format using ITK-SNAP software, version 3.8.0 (University of Pennsylvania, https://www.itksnap.org)^18^.

The converted files were uploaded to 3D Slicer software, version 5.2.2 (https://www.slicer.org/), an open-source 3D medical images viewing software^19^. 3D models of the scans were rendered using the “Segment editor” module, and the view layout was set to “four-up” mode to simultaneously display the 3D rendering along with the three orthogonal views (sagittal, coronal, axial). The 3D view facilitated confirmation of landmarks positions in the spatial space.

**Fig. 1:**
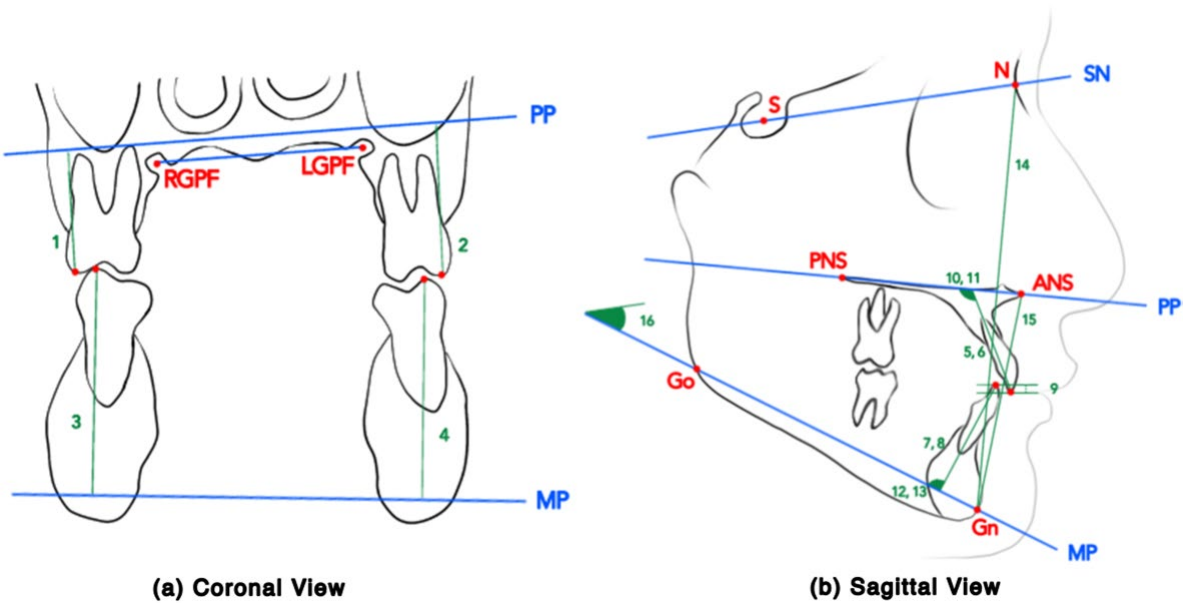
Cephalometric analysis. **(a)**: 1, (UR6-PP) The distance from the mesiobuccal cusp tip of the maxillary right first molar, measured perpendicularly to the palatal plane. 2, (UL6-PP) The distance from the mesiobuccal cusp tip of the maxillary left first molar, measured perpendicularly to the palatal plane. 3, (LR6-MP) The distance from the mesiobuccal cusp tip of the mandibular right first molar, measured perpendicularly to the mandibular plane. 4, (LL6-MP) The distance from the mesiobuccal cusp tip of the mandibular left first molar, measured perpendicularly to the mandibular plane. **(b)**: 5, (UR1-PP) The distance from the incisal edge of the maxillary right central incisor, measured along the root to the palatal plane. 6, (UL1-PP) The distance from the incisal edge of the maxillary left central incisor, measured along the root to the palatal plane. 7, (LR1-MP) The distance from the incisal edge of the mandibular right central incisor, measured along the root to the mandibular plane. 8, (LL1-MP) The distance from the incisal edge of the mandibular left central incisor, measured along the root to the mandibular plane. 9, (OB) The largest distance from the incisal edge of the maxillary central incisor to a horizontal reference line perpendicular to the incisal edge of the mandibular central incisor. 10, (∠UR1-PP) The angle between the long axis of the maxillary right central incisor and the palatal plane. 11, (∠UL1-PP) The angle between the long axis of the maxillary left central incisor and the palatal plane. 12, (∠LR1-MP) The angle between the long axis of the mandibular right central incisor and the mandibular plane. 13, (∠LL1-MP) The angle between the long axis of the mandibular left central incisor and the mandibular plane. 14, (AFH) The distance from Nasion to Gnathion. 15, (LAFH) The distance from the anterior nasal spine to Gnathion. 16, (MPA) The angle between the mandibular plane and Sella-Nasion line.

Cephalometric tracing and analysis were conducted by a single examiner using the “Markups” module. A total of 13 dental measurements (9 linear and 4 angular), along with 3 skeletal measurements, were selected for the analysis (Fig. 1). Landmark points were initially identified and approximated in the sagittal view by scrolling through the slices, then refined in the coronal and axial views. Final positional confirmation was done on the 3D model when visualization of the landmark was possible. This standardized viewing sequence was implemented to enhance consistency across the cases.

### Statistical analysis

Statistical Package for the Social Sciences software (SPSS, version 29.0.2.0, IBM Corporation, Armonk, NY, USA) was used to carry out statistical analysis.

Shapiro-Wilk test was performed to assess the distribution normality of the primary outcome. The test indicated a normal distribution of the data; hence, parametric statistics were applied. Descriptive statistics for the sample characteristics was done. The categorical variables (gender and Angle classification) were presented as frequencies and percentages, while the numerical variables, age, treatment duration, and tracing measurements were reported as means and standard deviations. To evaluate the intra-rater reliability of cephalometric tracing, 5 sets of scans were randomly selected for re-tracing and re-measuring by the same examiner with 2 weeks interval to compute Intraclass Correlation Coefficient (ICC). A comparison of the mean differences between T1 and T2 measurements was executed using paired t-test. Independent t-test and ANOVA were used to compare treatment changes based on gender and Angle classification, respectively. To control for false discovery rate associated with multiple comparison, the Benjamini-Hochberg procedure was applied independently to each of the three hypotheses, adjusting p-values for each set of tests, with a significance threshold set at FDR-adjusted *p* < 0.05. Pearson Correlation analysis of T2 values was conducted to examine the relationships among measurements and their association to the treatment outcome.

## Results

### Sample characteristics

After the initial screening, 49 AOB patients treated with Invisalign were included for review. 9 patients were excluded due to the following reasons: missing CBCT scans (*n* = 1), treatment involved extraction (*n* = 2), treatment involved fixed appliance with Invisalign (*n* = 5), single tooth anterior open bite on T1 scan (*n* = 1). Thus, the final sample size was 40.

The sample consisted of 11 (27.5%) male and 29 (72.5%) female patients who were treated between 2014 and 2022. The mean age was 32.78 ± 13.34 years, ranging from 12 to 63 years. Of the total sample, 31 patients had class I malocclusion, comprising 77.5% of the sample, 5 (12.5%) class II, and 4 (10%) class III patients. Successful correction of AOB was achieved in 100% of the sample, with a mean initial position of -2.41 ± 1.78 mm and an improvement of 4.26 ± 1.82 mm. The average treatment duration was 29 ± 13.5 months (Table 1).

**Table 1:** Sample characteristics.

Characteristic	*n*	%
Gender (male: female)	11 : 29	27.5 : 72.5
Angle Classification (I : II : III)	31 : 5 : 4	77.5 : 12.5 : 10
	Mean ± Standard deviation	
Age (years)	32.78 ± 13.34	
Treatment duration (months)	29 ± 13.5	

### Cephalometric analysis

#### Molar changes

ICC analysis showed that values for all variables ranged between 0.93 and 0.99, indicating high intra-rater measurement reliability.

The maxillary right first molar showed a statistically significant reduction in vertical height relative to the palatal plane by 0.76 ± 1.33 mm (adjusted *p* = 0.006) compared to its initial position, whereas the left molar decreased by 0.33 ± 1.30 mm, which was not statistically significant (adjusted *p* = 0.24). When both sides were combined as overall maxillary molar vertical height, a significant decrease of 0.53 ± 1.17 mm (adjusted *p* = 0.021) was observed (Table 2). In contrast, the mandibular first molars exhibited a slight, statistically insignificant increase—0.15 ± 1.36 mm on the right, 0.18 ± 1.56 mm on the left, and 0.17 ± 1.41 mm when combined (adjusted *p* = 0.489, 0.489, 0.489) (Table 2).

#### Incisal changes

The maxillary incisors showed an increase in vertical position relative to the palatal plane, measuring 0.67 ± 1.81 mm on the right and 0.48 ± 1.66 mm on the left. Although the right-side change was statistically significant (unadjusted *p* = 0.026), it only approached significance after correction for multiple comparison (adjusted *p* = 0.052). Similarly, when both sides were combined, the overall maxillary incisor vertical position increased significantly by 0.57 ± 1.70 mm (unadjusted *p* = 0.042); however, it did not retain statistical significance following correction (adjusted *p* = 0.07) (Table 2). The mandibular incisors exhibited minimal changes in vertical position—0.34 ± 2.00 mm on the right (unadjusted *p* = 0.297), 0.40 ± 1.95 mm on the left (unadjusted *p* = 0.204), and 0.36 ± 1.96 mm when combined (unadjusted *p* = 0.260)—none of which reached statistical significance (Table 2). A significant reduction in the inclination of the maxillary incisors was noted, with a mean decrease of 4.04 ± 6.99° on the right and 4.91 ± 7.22° on the left (adjusted *p* = 0.003, 0.003). Likewise, the mandibular incisors also demonstrated a reduction in inclination, with a decrease of 5.64 ± 7.63° on the right and 5.16 ± 7.13° on the left (adjusted *p* = 0.003, 0.003) (Table 2).

#### Skeletal changes

AFH showed an insignificant decrease of 0.68 ± 2.71 mm, both before and after correction of *p*-value (unadjusted *p* = 0.124). LAFH and MPA demonstrated significant reductions, with decreases of 0.72 ± 2.17 mm and 0.96 ± 2.63°, respectively (unadjusted *p* = 0.043, 0.026). However, neither of the adjusted *p*-values reached statistical significance (adjusted *p* = 0.065, 0.065) (Table 2).

**Table 2:** Cephalometric values, treatment changes, and significance.

	Variable	T1	T2	T2-T1	Unadjusted *P*-value	Adjusted *P*-value
Molar	UR6-PP	25.70 ± 3.25	24.94 ± 3.10	-0.76 ± 1.33	< 0.001*	0.006*
	UL6-PP	24.99 ± 3.45	24.67 ± 3.27	-0.33 ± 1.30	0.120	0.240
	∑U6-PP	25.36 ± 3.26	24.84 ± 3.08	-0.53 ± 1.17	0.007*	0.021*
	LR6-MP	32.85 ± 3.93	33.00 ± 3.89	0.15 ± 1.36	0.489	0.489
	LL6-MP	32.42 ± 3.71	32.60 ± 3.62	0.18 ± 1.56	0.466	0.489
	∑L6-MP	32.64 ± 3.77	32.80 ± 3.71	0.17 ± 1.41	0.462	0.489
Incisor	UR1-PP	32.13 ± 3.98	32.79 ± 3.49	0.67 ± 1.81	0.026*	0.052
	UL1-PP	32.28 ± 4.09	32.75 ± 3.63	0.48 ± 1.66	0.078	0.111
	∑U1-PP	32.21 ± 3.99	32.77 ± 3.53	0.57 ± 1.70	0.042*	0.070
	LR1-MP	37.68 ± 4.22	38.02 ± 4.51	0.34 ± 2.00	0.297	0.297
	LL1-MP	37.65 ± 4.19	38.05 ± 4.45	0.40 ± 1.95	0.204	0.255
	∑L1-MP	37.69 ± 4.19	38.05 ± 4.45	0.36 ± 1.96	0.260	0.289
	∠UR1-PP	113.11 ± 8.79	109.07 ± 7.29	-4.04 ± 6.99	< 0.001*	0.003*
	∠UL1-PP	113.97 ± 9.41	109.07 ± 7.37	-4.91 ± 7.22	< 0.001*	0.003*
	∠LR1-MP	96.63 ± 9.77	90.99 ± 7.57	-5.64 ± 7.63	< 0.001*	0.003*
	∠LL1-MP	96.22 ± 9.33	91.06 ± 7.52	-5.16 ± 7.13	< 0.001*	0.003*
Skeletal	AFH (mm)	117.11 ± 7.46	116.44 ± 8.00	-0.68 ± 2.71	0.124	0.124
	LAFH (mm)	68.34 ± 7.01	67.62 ± 7.01	-0.72 ± 2.17	0.043*	0.065
	MPA (°)	36.32 ± 5.59	35.36 ± 5.59	-0.96 ± 2.63	0.026*	0.065

Values are described as mean and standard deviation.

Paired t-test was performed to compare mean T1 and T2, * statistical significance at *p* < 0.05.

Benjamini-Hochberg procedure was performed to control False Discovery Rate, * statistical significance at FDR-adjusted *p* < 0.05.

### Other analyses

Independent t-test and ANOVA revealed that there are no considerable differences in treatment changes based on gender or Angle classification, except for ∑L1-MP. Males showed more increase in the mandibular incisors’ vertical height of 1.59 ± 1.41 mm compared to females (-0.48 ± 1.37 mm, adjusted *p* = 0.009).

**Fig. 2:**
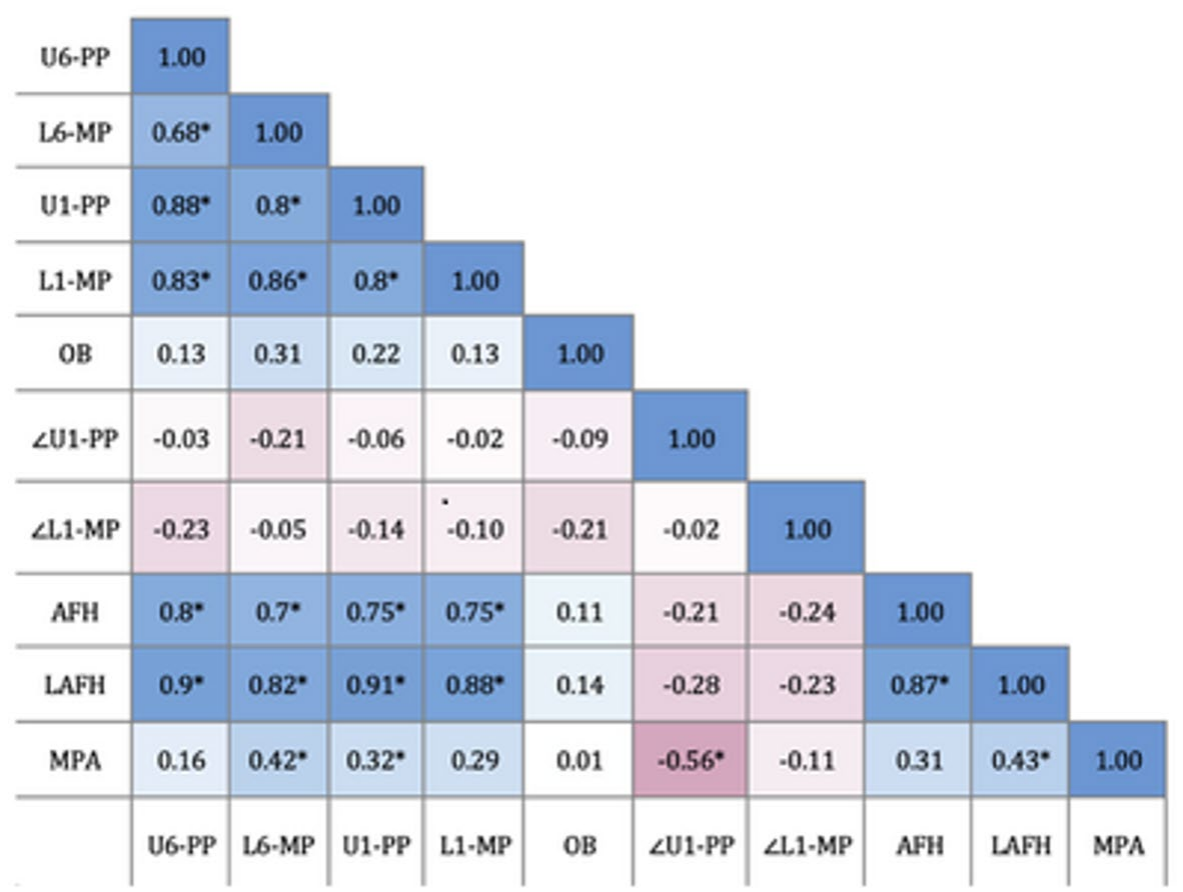
Heatmap illustrating Pearson Correlation Coefficients (r values) of T2 dental and skeletal variables. Positive correlations are represented in blue tones and negative correlations in red tones, with color intensity indicating the strength of the correlation, * statistical significance at *p* < 0.05.

Pearson correlation analysis revealed that overbite showed no significant correlation with any of the measurements. However, the T2 vertical position of both the maxillary and mandibular molars showed a strong correlation with AFH and LAFH. Additionally, MPA demonstrated a moderate but significant correlation with LAFH (Fig. 2).

## Discussion

In this retrospective cohort study, the aim was to quantify the dental and skeletal changes that contribute to the correction of AOB using Invisalign therapy. This was accomplished through cephalometric analysis of 40 sets of pre-treatment and post-treatment CBCT scans. Open-source software programs—ITK-SNAP and 3D Slicer—were employed for image processing and landmark tracing. The analysis included 13 dental and 3 skeletal measurements, recorded in a systematic approach to improve reliability and consistency. This was demonstrated by high intra-class correlation coefficients for all measurements, ranging from 0.93 to 0.99. Similar findings have been reported in previous studies, which also observed higher reliability in multi-planar reconstruction views, although variability was noted for certain landmarks^13,20^.

Our sample demonstrated successful AOB correction, with a final overbite of 1.85 ± 0.89 mm, despite the severity of the pre-treatment open bite being − 2.41 ± 1.78 mm. This value was higher than those reported by Khosravi et al., Moshiri et al., Garnett et al., Harris et al., Steele et al., and Suh et al.; nevertheless, the amount of overbite change (4.26 ± 1.82 mm) exceeded the findings of these studies^8–10,21,22^.

The vertical position of the maxillary and mandibular molars was considered as the primary outcome of this study. To our knowledge, no previous research has utilized CBCT imaging to assess vertical changes of both the right and left molars independently, rather than tracing the average or the most anterior tooth. The analysis showed unequal intrusion between the maxillary right and left sides, which may be attributed to the lower initial level of the right side compared to the left (R: 25.70 ± 3.25 mm; L: 24.99 ± 3.45 mm), thus necessitating more intrusion on that side.

When both sides were combined, the mean of total maxillary molar intrusion was 0.53 ± 1.17 mm with a significant difference compared to the initial position. This was slightly greater than the highest mean intrusion reported by Harris et al. (– 0.47 ± 0.59 mm)^9^. Moreover, when Suh et al. stratified their sample based on Angle classification, Class II patients exhibited the greatest maxillary molar intrusion (− 0.56 ± 0.51 mm); however, our sample showed no differences across Angles Classes^22^. In contrast, the vertical position of the mandibular molars remained unchanged, which differs from the findings of Moshiri et al. and Harris et al., who reported significant mandibular molar intrusion. Overall, these findings suggest that Invisalign treatment either resulted in maxillary and mandibular molar intrusion or maintained their vertical positions.

The second objective of this study was to evaluate the vertical position and inclination of the incisors. The amount of extrusion observed in the maxillary incisors was insignificant after the adjustment for False Discovery Rate, while both maxillary and mandibular incisors exhibited significant retroclination. This contrasts Moshiri et al., who reported significant extrusion of only the lower incisors, and Khosravi et al., who noted extrusion in both arches; however, neither study assessed incisor inclination, whereas significant extrusion and retroclination were observed by other authors^9,10,23^. Suh et al. found that while maxillary incisor extrusion occurred across all Angle classifications, Class II patients demonstrated greater extrusion and retroclination compared to Class I and III. Similarly, Class III cases showed more mandibular incisor extrusion and retroclination^22^. Such Class-based variations were not detected in the present sample, potentially due to the smaller sample size and the unequal distribution of Angle classifications.

Regarding skeletal changes, a decreasing trend was observed in AFH, LAFH, and MPA, consistent with previous studies that reported significant molar intrusion. However, none of these reductions remained statistically significant after correction for multiple comparisons, although a strong correlation was found between AFH and LAFH and the vertical position of both maxillary and mandibular molars.

This study has several limitations. First, the retrospective design limited control over treatment planning variables such as IPR, expansion, and elastic use. Second, measurements were recorded by a single examiner, and while this avoids inter-examiner variability, it may increase the risk of measurement bias. The absence of a control group further restricts the ability to attribute observed changes solely to clear aligner therapy. Lastly, while statistical corrections for multiple comparisons were applied, the relatively small sample size may limit the power to detect changes in some variables that were documented as significant.

## Conclusions

- Clear aligner therapy demonstrated effective anterior open bite correction in all cases through dental movements, primarily maxillary molar intrusion and retroclination of maxillary and mandibular incisors.
- Clear aligner therapy is effective in controlling the vertical dimension.
- Further prospective studies with larger sample sizes and comparison groups are needed to validate these findings and to comprehensively assess changes across the entire posterior dentition and skeletal structures.

## Data Availability

The datasets generated during and/or analysed during the current study are available from the corresponding author on reasonable request.
